# The molecular mechanisms and intervention strategies of mitophagy in cardiorenal syndrome

**DOI:** 10.3389/fphys.2022.1008517

**Published:** 2022-10-24

**Authors:** Mengying Yao, Yong Liu, Mengjia Sun, Shaozong Qin, Wang Xin, Xu Guan, Bo Zhang, Ting He, Yinghui Huang

**Affiliations:** ^1^ Department of Nephrology, The key Laboratory for the Prevention and Treatment of Chronic Kidney Disease of Chongqing, Chongqing Clinical Research Center of Kidney and Urology Diseases, Xinqiao Hospital, Army Medical University (Third Military Medical University), Chongqing, China; ^2^ Department of Cardiology, Institute of Cardiovascular Diseases of PLA, The Second Affiliated Hospital, Army Medical University (Third Military Medical University), Chongqing, China

**Keywords:** CRS, CKD, CVD, targeted-therapy, mitophagy

## Abstract

Cardiorenal syndrome (CRS) is defined as a disorder of the heart and kidney, in which acute or chronic injury of one organ may lead to acute or chronic dysfunction of the other. It is characterized by high morbidity and mortality, resulting in high economic costs and social burdens. However, there is currently no effective drug-based treatment. Emerging evidence implicates the involvement of mitophagy in the progression of CRS, including cardiovascular disease (CVD) and chronic kidney disease (CKD). In this review, we summarized the crucial roles and molecular mechanisms of mitophagy in the pathophysiology of CRS. It has been reported that mitophagy impairment contributes to a vicious loop between CKD and CVD, which ultimately accelerates the progression of CRS. Further, recent studies revealed that targeting mitophagy may serve as a promising therapeutic approach for CRS, including clinical drugs, stem cells and small molecule agents. Therefore, studies focusing on mitophagy may benefit for expanding innovative basic research, clinical trials, and therapeutic strategies for CRS.

## 1 Introduction

Accumulated evidence demonstrated the increasing incidence of cardiac and renal failure, and the co-existence of these two diseases is characterized with an extremely poor prognosis. Cardiorenal syndrome (CRS) has been introduced to emphasize the crosstalk between the kidney and heart in the context of acute or chronic disease (Ronco et al., 2010). CRS is mainly divided into five types according to clinical etiology. Herein, we principally focus on Cardiorenal syndrome type 4 (CRS4), since cardiovascular disease (CVD) is the most common complication, as well as the leading cause (over 50%) of death in chronic kidney disease (CKD) patients (Granata et al., 2016). It is estimated that up to 30% of CKD patients suffer from heart failure (HF), while approximate 40–50% of patients with HF also accompanies by CKD (Tuegel and Bansal, 2017). In addition, the risk of HF is reported to be much higher in patients with an estimated glomerular filtration rate (eGFR) < 60 ml/min per 1.73  m^2^, especially in those dialysis patients (Tuegel and Bansal, 2017) (Bansal et al., 2017). However, the underlying mechanisms by which CKD patients suffer increased susceptibility to CVD or patients with CVD often coexist with CKD remain elusive. Therefore, further clarifying the mechanism of CRS4 and developing efficient treatments are urgently needed.

**FIGURE 1:**
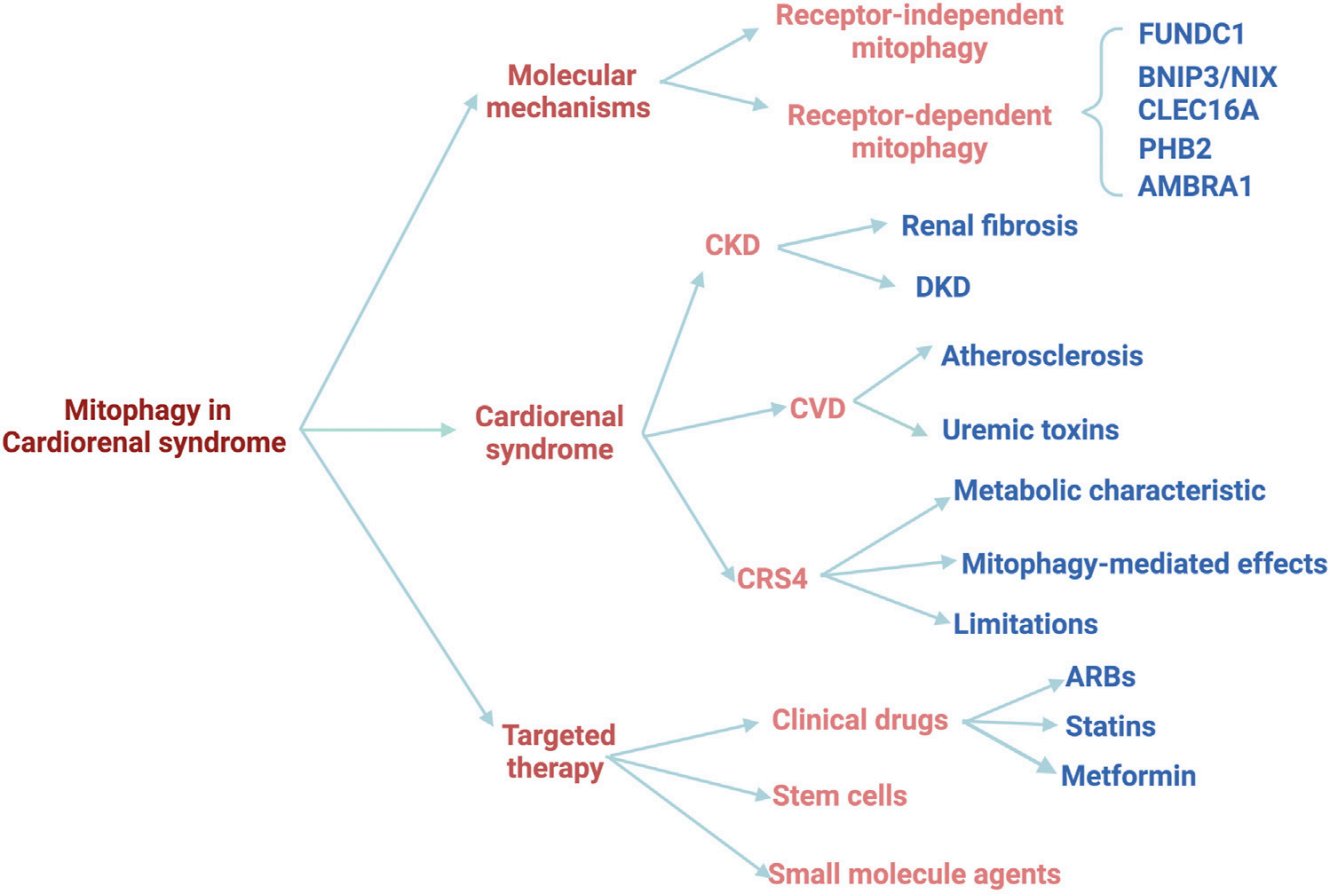
The schematic diagram of the review.

The heart and kidney are the top two organs with the most mitochondria in the human body. Mitochondria play a crucial role in the pathophysiological processes in these two organs, including energy metabolism remodeling, reactive oxygen species (ROS) production, apoptosis, and signaling transduction, while mitochondrial injury is a common feature of both CKD and CVD. Of note, the damaged mitochondria will release a large amount of ROS, which will further aggravate mitochondrial damage, forming a vicious circle. Mitochondria-specific autophagy (mitophagy) is an essential and fundamental process for maintaining mitochondrial health in various types of cells, including cardiomyocytes and renal cells. Mitophagy preserves mitochondrial homeostasis and mitochondrial quality control through selectively eliminating damaged mitochondria. Recently, the role and mechanism of mitophagy have been extensively investigated in multiple disease models (Bravo-San Pedro et al., 2017) (Kerr et al., 2017) (Lou et al., 2020)), and numerous studies have confirmed the association between mitophagy and CKD as well as CKD-associated CVD (Huang et al., 2020b) (Bhargava and Schnellmann, 2017). Therefore, targeting mitophagy might ameliorate both CKD and CVD, which sheds new light that mitophagy may serve as a promising therapeutic target for CRS4. In this review, we will summarize the important findings of mitophagy in CKD and CVD, especially the crucial roles and underlying mechanisms of mitophagy activation in CRS, and highlight the pharmacologic modulation of mitophagy as a potential therapeutic strategy (Figure 1).

### 1.1 Molecular mechanisms of mitophagy

**FIGURE 2:**
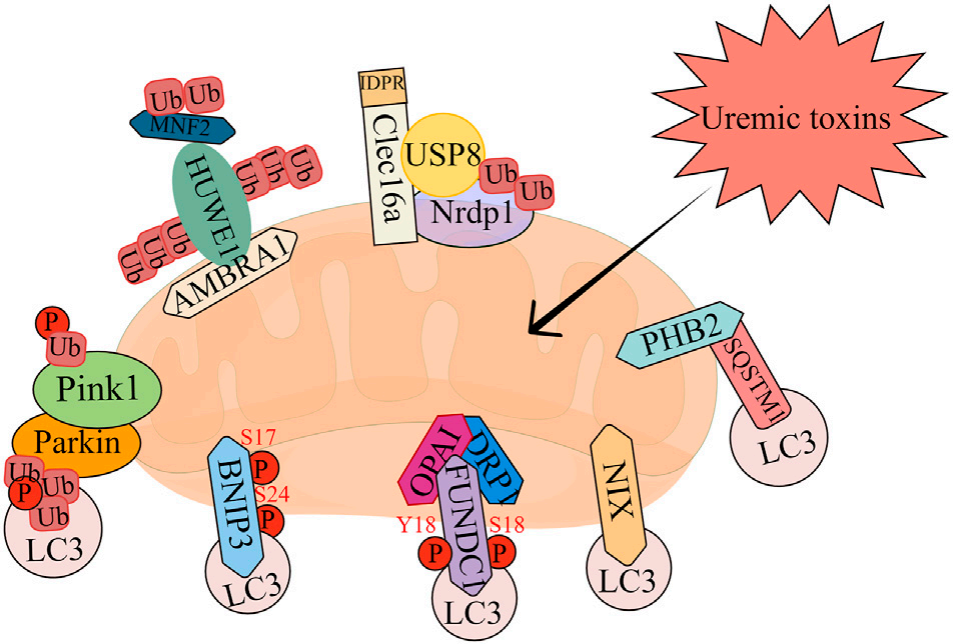
The overview of mitophagy pathways.

The molecular mechanisms of mitophagy have been intensively investigated in multiple species. There are mainly two types of mitophagy, according to the signal pathway, including receptor-dependent and -independent mitophagy. In addition to the traditional signal pathways, there are also some newly reported genes that can trigger mitophagy, such as C-type lectin domain containing 16A (Clec16a) and Prohibitin 2 (PHB2). Here we summarize the current important pathways involved in mitophagy (Figure 2).

### 1.2 Receptor-independent mitophagy

Most of the studies investigating the role of mitophagy in physiological or pathological conditions focus on the PTEN Induced Kinase 1 (PINK1)-Parkin RBR E3 Ubiquitin Protein Ligase (Parkin) pathway, which is the most classic mitophagy signaling. PINK1 is a serine/threonine kinase that acts as a molecular sensor mainly through recruiting and activating Parkin. Under normal conditions, PINK1 enters mitochondrial inner membrane *via* its mitochondria-targeting sequence with the help of translocase of the outer mitochondrial membrane (TOM) and the endomembrane translocation enzyme TIM23 complex. After entering mitochondrial inner membrane, PINK1 is cut by mitochondrial processing peptidase, and mitochondrial protease presenilin associated rhomboid like (PARL) to produce a 52 kD PINK1 with N-terminal deletion (Deas et al., 2011). PINK1 is then released into the cytoplasm, where it targets N-degron type-2 E3 ubiquitin ligase and is degraded by ubiquitin proteasome system (Yamano and Youle, 2013). This ordered input and degradation of PINK1 keep PINK1 at a very low level in healthy mitochondria. But in pathological condition, PINK1 cannot be imported into the mitochondrial inner membrane, and then accumulate in the mitochondrial outer membrane, which is an important step for activating Parkin. Once entering the mitochondrial inner membrane, Parkin will enhance the ubiquitination of mitochondrial outer membrane proteins, thereby recruiting autophagosomes to the damaged mitochondria. Importantly, Parkin can also be recruited to depolarized mitochondria and drive mitophagy even without PINK1 (Ni et al., 2015). Once recruited into mitochondria, Parkin will ubiquitinate several mitochondrial outer membrane proteins, including mitochondrial fusion proteins mitofusin-1 (Mfn1), mitofusin-2 (Mfn2), and the voltage-dependent anion channel (VDAC), to initiate mitophagy. Mfn1, Mfn2 and dynamin-related protein 1 (DRP1) are key participants in controlling mitochondrial dynamics and coordinating mitochondrial network connections and activities (Mishra and Chan, 2016). Ubiquitination and proteasome degradation of Mfn1, Mfn2 or DRP1 lead to mitochondrial fission or fusion (Chan et al., 2011), but the function remains during the fission process, thereby promoting mitophagy (Tanaka et al., 2010). It has also been reported that PINK1 can phosphorylate Mfn2 and then act as a Parkin receptor to clear damaged mitochondria (Chen and Dorn, 2013). It has also been proved that VDAC1 plays a vital role in the initiation of mitophagy *via* interacting with Parkin and participating in Parkin recruitment and ubiquitination (Shoshan-Barmatz et al., 2018).

### 1.3 Receptor-dependent mitophagy

Receptor-dependent mitophagy is another kind of recognition initiation mechanism, which depends on the LC3-interacting region (LIR) proteins such as FUN14 domain-containing 1 (FUNDC1), Bcl-2/adenovirus E1B 19 kDa interacting protein (Bnip3) and Bnip3 like (Bnip3L/NIX) on the impaired mitochondrial outer membrane.

## 2 FUN14 domain-containing 1

FUNDC1 is a recently discovered mitochondrial protein. FUNDC1-mediated mitophagy is regulated by reversible phosphorylation. Under physiological conditions, the LIR of FUNDC1 is phosphorylated by casein kinase 2 (CK2) at Ser13 and Src protein-tyrosine kinase at Tyr18, thus inhibiting its interaction with LC3. Therefore, it has almost no mitophagy activity under normal conditions (Kuang et al., 2016). However, under the stimulation of hypoxia or Carbonyl cyanide 4-trifluoromethoxy phenylhydrazone (FCCP), the dephosphorylation of Ser13 and Tyr18 enhance the interaction between FUNDC1 and LC3, leading to mitophagy. (Liu et al., 2014). Moreover, under pathological conditions, FUNDC1 is phosphorylated by unc-51 like autophagy activating kinase 1 (ULK1) at Ser17 to promote its binding to LC3, contributing to the occurrence of mitophagy (Wu et al., 2014). Recently, a mitochondria ubiquitin ligase, membrane associated ring-CH-type finger 5 (MARCH5), has also been proved to be involved in FUNDC1-mediated mitophagy. The aggregated MARCH5 interacts with and degrades FUNDC1 through Parkin-mediated ubiquitination, resulting in a decrease in the binding of FUNDC1 to LC3 and a negative regulation of mitophagy, maintaining a certain level of mitophagy and cell homeostasis (Chen Z. et al., 2017). In addition, FUNDC1 can also interact with mitochondrial fusion and fission proteins (DRP1 and OPA1) to regulate mitochondrial dynamics and mitophagy. (Chen et al., 2016). Under hypoxic conditions, the binding of FUNDC1 to DRP1 is enhanced, which leads to the recruitment of DRP1 to mitochondria and subsequent mitochondrial division, thereby promoting the occurrence of mitophagy (Wu et al., 2016).

## 3 BNIP3/NIX

Bnip3 is initially considered to be a mitochondrial pro-apoptotic protein (Chen et al., 1997). NIX share homology with Bnip3 (53%–56% amino acid sequence identity) (Matsushima et al., 1998). Recent studies have shown that Bnip3 and its homolog NIX can specifically activate mitophagy through directly interacting with LC3 *via* their LIR motifs on autophagosomes (Hanna et al., 2012) (Novak et al., 2010). Bnip3 is usually expressed as an inactive monomer in cytosol. After stress signal, it forms a stable homodimer *via* its C-terminal transmembrane domain (TM) and integrates into the mitochondrial outer membrane. C-terminal NIX phosphorylation and its consequent dimerization loss could decrease the induction of NIX-mediated mitophagy. (Marinkovic et al., 2021).

### 3.1 Mitophagy-related novel genes

#### 3.1.1 Clec16a

Clec16a has been previously identified as a susceptibility gene for type 1 diabetes, multiple sclerosis, and adrenal dysfunction, but its physiological function remains unclear (Hakonarson et al., 2007) (International Multiple Sclerosis Genetics, 2009) (Skinningsrud et al., 2008). Recent studies reported that Clec16a is a membrane-associated endosome protein which can interact with the E3 ubiquitin ligase Neuregulin receptor degradation protein-1 (Nrdp1) (Soleimanpour et al., 2014). Loss of Clec16a leads to an increase in the Nrdp1 target Parkin, a master regulator of mitophagy. Islets from mice with pancreas-specific deletion of Clec16a have abnormal mitochondria with reduced oxygen consumption and ATP concentration (Soleimanpour et al., 2014).

#### 3.1.2 Prohibitin 2

PHB2 is a conserved inner mitochondrial membrane (IMM) protein which plays an important role in multiple cellular processes, including mitochondrial dynamics (Artal-Sanz and Tavernarakis, 2009). PHB2, as a mitophagy receptor, is involved in targeting mitochondria for mitophagic degradation. Upon mitochondrial depolarization and proteasome-dependent outer membrane rupture, PHB2 interacts with the autophagosomal membrane-associated protein LC3 *via* a LIR domain. (Wei et al., 2017). On the other hand, PHB2 forms a ternary protein complex with sequestosome 1 (SQSTM1) and LC3, resulting in the loading of LC3 into damaged mitochondria (Xiao et al., 2018). Further, PHB2 depletion destabilizes PINK1 in mitochondria, thereby preventing the mitochondrial recruitment of Parkin, ubiquitin and optineurin (OPTN). Therefore, PHB2 knockdown inhibits mitophagy, while overexpression of PHB2 alleviates mitochondrial dysfunction by ameliorating pyrin domain-containing protein 3 (NLRP3)-induced inflammatory pathways. (Xu et al., 2019).

#### 3.1.3 Autophagy and beclin 1 regulator 1

Autophagy and beclin 1 regulator 1 (AMBRA1) is a multifunctional protein with well-known autophagic and mitophagic functions (Di Rienzo et al., 2021) (Strappazzon et al., 2020) (Strappazzon et al., 2015) (Zhou et al., 2020). Upon mitochondrial depolarization, the pro-autophagic protein AMBRA1 is recruited to the OMM and interacts with PINK1 and ATPase family AAA domain containing 3A (ATAD3A), a transmembrane protein that mediates mitochondrial import and degradation of PINK1. Emerging evidences have reported that AMBRA1 regulates mitophagy though other two critical steps. Upon mitophagy stimulation, AMBRA1 translocates from cytosol to mitochondria, acting as a cofactor for HUWE1 E3 ubiquitin ligase, favoring its binding to its substrate Mfn2 and subsequent targeting to the proteasome. This event is crucial and required for AMBRA1-induced mitophagy. In the second step, similar to other mitochondrial receptors, AMBRA1 is phosphorylated at S1014 in its LIR motif, and then interacts with LC3, ultimately leading to the engulfment of damaged mitochondria by autophagosomes. This post-translational modification is controlled by IKKα kinase upon mitophagy stimulation. (Di Rita et al., 2018).

## 4 Mitophagy in cardiorenal syndrome type 4

**FIGURE 3:**
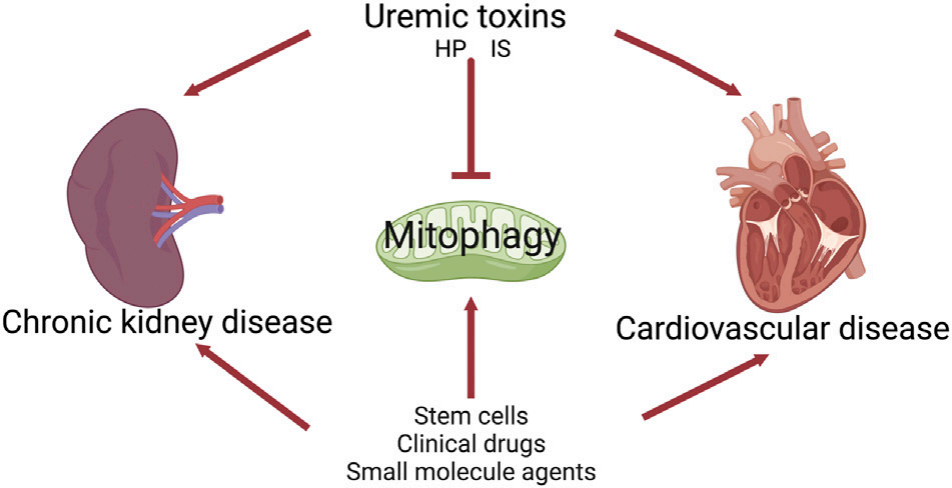
The role of mitophagy in cardiorenal syndrome type 4.

CRS4 is defined as chronic cardiac damage caused by CKD, including left ventricular hypertrophy, cardiac insufficiency, and/or increased adverse cardiovascular events. In the present study, we speculate that modulating mitophagy may serve as one of the promising therapeutic strategies for the progression of CRS4 (Figure 3).

### 4.1 Mitophagy in chronic kidney disease

The kidney plays a vital role in maintaining normal function of the body and requires a large amount of energy, which is provided by mitochondrial oxidative metabolism. Therefore, maintaining mitochondrial homeostasis and quality control is critical for renal function. Previous studies have shown that mitochondrial damage and dysfunction play a pivotal role in the progression of CKD (Aparicio-Trejo et al., 2020) (Bai et al., 2019) (Fujimura et al., 2020) (Gu et al., 2021) (Zhu et al., 2021) (Li X. et al., 2020). Given that mitophagy is a key mechanism in the control of mitochondrial quality, targeting mitophagy may be an important therapeutic approach in the pathogenesis and progression of CKD.

### 4.2 Renal fibrosis

As reported, mitophagy-related genes PINK1, Mfn2 and Parkin were decreased in human patients and animal models with renal fibrosis (Chen et al., 2018). Pink1^−/−^ and Parkin^−/−^ mice exhibited suppression of mitophagy, impaired mitochondrial homeostasis, enhanced recruitment of profibrotic M2 macrophages, and aggravated renal fibrosis (Bhatia et al., 2019). In adenine-induced CKD mice, elimination of Mfn2 in myeloid cells resulted in decreased mitophagy and increased recruitment of macrophages to the kidney, enhanced the macrophage-derived fibrotic response, and eventually deteriorated renal function. However, Li et al. found that the Pink1-Parkin pathway was activated in UUO models, while the deletion of PINK1-Parkin signaling aggravated renal tubular injury and subsequent renal fibrosis, which could be alleviated by using a mitochondrial-targeted antioxidant, MitoTEMPO.

### 4.3 Chronic kidney disease

Diabetes is the leading cause of CKD in both developing and developed countries (Webster et al., 2017b). It is well known that mitophagy is inhibited under high glucose conditions (Audzeyenka et al., 2021). For example, the expression levels of mitophagy receptors, OPTN and PINK1, were significantly decreased in human diabetic specimens (Chen et al., 2018). Of note, overexpression of OPTN significantly enhanced the activation of mitophagy by inhibiting NLRP3 inflammasome, retarded cell senescence and alleviated diabetic nephropathy, while OPTN silencing markedly inhibited high glucose-induced mitophagy. Therefore, OPTN-mediated mitophagy plays a crucial regulatory role in high glucose-induced inflammation and renal tubular senescence in diabetic nephropathy. Mdivi-1, a mitochondrial fission/mitophagy inhibitor, accelerated renal tubular senescence, while Torin1, an autophagy/mitophagy agonist, inhibited cell senescence (Chen et al., 2018). In addition to renal tubular cells, podocyte injury plays a central role in the development of diabetic nephropathy. Therefore, protecting podocytes delayed the progression of diabetic nephropathy (Jiang et al., 2020) (Kong et al., 2020) (Zheng T. et al., 2022). Recombinant human PGRN protein activated Sirt1-PGC1α-Foxo1 signaling to improve mitophagy and maintain mitochondrial homeostasis in podocytes, which provides a new therapeutic target for DKD. Meanwhile, non-coding RNAs are also closely related to mitophagy in diabetes. Silencing lncRNA SNHG17 alleviated DKD by activating Parkin-dependent mitophagy *via* regulating the degradation of Mst1. The above studies demonstrate that mitophagy possess considerable protective effect in DKD and targeting mitophagy may provide new concepts for the treatment of DKD.

### 4.4 Chronic kidney disease-related cardiovascular disease

Cardiac and renal hemodynamic regulation is a dynamic system. The kidney receives about 25% of cardiac output, thus the continuous decline of cardiac output leads to insufficient renal artery perfusion. As reported, CVD is one of the most common complications of CKD. CVD, including coronary artery disease, congestive HF, arrhythmias, and sudden cardiac death, is the leading cause of death in CKD patients (Schefold et al., 2016) (Whitman et al., 2012) (Narala et al., 2013). Notably, mitochondrial damage-mediated CVD is not only the result of renal dysfunction, but also contributing factor to renal damage, promoting the progression of CKD and eventually forming a vicious circle (Dickhout et al., 2011). Therefore, timely removal of damaged mitochondria through mitophagy to maintain mitochondrial homeostasis is particularly important for the normal contractile function of the myocardium.

It has been reported that general autophagy and mitophagy transiently increased in cardiac tissue during the early stages of HF, and mitophagy levels significantly decreased during the chronic phase of TAC. Meanwhile, Previous studies have shown that the expression level of PINK1 was reduced in the tissues of patients with end stage HF (Billia et al., 2011), indicating that mitophagy is inhibited in the process of HF. Subsequent studies showed that PINK1 silenced or knocked out mice were susceptible to reperfusion injury and stress overload, resulting in HF (Siddall et al., 2013). Moreover, cytoplasmic p53 bound to Parkin and interfered with its translocation to damaged mitochondria. Inhibition of mitophagy led to increased Adriamycin-induced cardiomyocyte injury, demonstrating that inhibition of mitophagy contributes to mitochondrial dysfunction and accelerates the progression of HF. In addition, numerous studies have shown that moderate increase of mitophagy level can repress the progression of HF (Dhingra and Kirshenbaum, 2022) (Abudureyimu et al., 2020) (Li Q. et al., 2022) (Yu et al., 2018), (Shirakabe et al., 2016)). Wang et al. reported that overexpression of AMPKα2 phosphorylated PINK1 at Ser495 site, activated the PINK1-Parkin-SQSTM1 pathway, and eliminated the impaired mitochondria in HF. In addition, the expression of DRP1 significantly increased from 2 to 5 days in the stress overload model, which was consistent with the activation period of mitophagy. Meanwhile, DRP1 deletion led to inhibition of mitophagy, aggravating the stress-induced cardiac dysfunction. Although proper mitophagy is protective during HF, excessive mitophagy may exacerbate cellular damage. Another study demonstrated that abnormal upregulation of Bnip3 destroyed the mitochondrial integrity, leading to the increase of superoxide production and the release of pro-apoptotic factors (such as cytochrome c and apoptosis-inducing factor (AIF)) in the process of cardiac ischemia-reperfusion injury (IRI) (Hamacher-Brady et al., 2007). Subsequently, Abhinav et al. reported that ventricular remodeling and dysfunction were significantly alleviated in Bnip3 knockout (KO) mouse model after myocardial IRI. NIX was also reported to enhance cardiomyocyte apoptosis and lead to pathological myocardial remodeling (Yussman et al., 2002). The above data collectively suggested that moderate mitophagy removes damaged mitochondria, reduces mitochondrial ROS and maintains myocardial homeostasis. However, excessive mitophagy leads to excessive mitochondrial clearance, leading to insufficient energy production to preserve myocardial consumption. Since mitophagy has dual roles, how to control the degree of mitophagy in myocardial injury needs further investigation.

### 4.5 Atherosclerosis

The level of mitophagy is highly correlated with oxidized low-density lipoprotein (OX-LDL), which is a key factor in promoting the development of atherosclerosis. *In vitro* studies showed that the activation of mitophagy inhibited the apoptosis of human VSMC induced by OX-LDL (Swiader et al., 2016). Enhance expression of PINK1 improved the level of mitophagy, thereby increasing its protective effect on VSMC, while silencing PINK1 reversed this effect (Swiader et al., 2016) (Docherty et al., 2018)). On the contrary, excessive mitophagy overtly consumed mitochondrial mass, leading to an energy shortage and mitochondrial dysfunction. Such as, in ApoE^−/−^ mice induced atherosclerosis, apelin-13 enhanced Pink1/Parkin-mediated mitophagy *via* activating p-AMPKα, therefore, induced VSMC proliferation to aggravate the development of atherosclerotic lesions (He et al., 2019). In addition, phosphatase and tensin homolog (PTEN) promoted endothelial cell apoptosis by inhibiting mitophagy (Li P. et al., 2020). Thus, precisely modulating mitophagy is a challenging and promising therapeutic approach for atherosclerosis.

### 4.6 Uremic toxins

#### 4.6.1 Indoxyl sulfate

The accumulation of uremic toxins in blood and tissues is associated with the progression of CKD and its complications, including CVD. Indoxyl sulfate (IS) has shown nephrotoxic effects through generation of ROS (Owada et al., 2008), depletion of anti-oxidative systems (Shimizu et al., 2011), and induction of fibrosis and inflammation. Our previous studies have shown that IS inhibited the DRP1-mediated mitophagy flux, whereas DRP1 overexpression attenuated IS-induced mitophagy inhibition and cell damage (Huang et al., 2020b). Moreover, administration of an oral adsorbent for IS, AST-120, attenuated IS-induced DRP1 reduction and mitophagy impairment in CKD mice (Huang et al., 2020b). IS also implicated with CVD, vascular calcification, vascular stiffness, and congestive HF in patients with ESRD (Hung et al., 2017). *In vitro* studies have shown that IS directly induced endothelial dysfunction, an early marker of atherosclerosis (Hung et al., 2017). Collectively, these reports imply that IS may inhibit mitophagy during CRS4.

#### 4.6.2 High phosphate

Vascular calcification refers to the ectopic deposition of calcium and phosphorus complexes in the vascular wall, which is a common pathological manifestation in CKD patients. Disruption of mineral homeostasis and high phosphate (HP) levels are the main determinants of vascular calcification in CKD. (Voelkl et al., 2018) (Alesutan et al., 2017). As reported, increased levels of calcium phosphate products associated with the development of vascular calcification in CKD (Chen J. et al., 2017; Liu et al., 2018; Voelkl et al., 2019) (Cozzolino et al., 2019), which would lead to increased vascular stiffness, the main contributing factor to CVD in dialysis patients (Galetta et al., 2005) (Melamed et al., 2006). Interestingly, in calcific aortic stenosis, the increase of lactic acid was accompanied by the increase of mitophagy and mitochondrial damage, but the use of RAPI to enhance mitophagy can still alleviate valve calcification (Morciano et al., 2021). Our recent study demonstrated that HP-induced downregulation of PGC1α contributed to CRS4 *via* upregulating IRF1, while restoring PGC1α expression ameliorated energy metabolism disorders and HF (Huang et al., 2020a).

### 4.7 Metabolic characteristics of CRS4

The metabolic characteristics of CRS4 is characterized by a reduction of FAO and OXPHOS, accompanied by a compensatory increase in glycolysis, which were observed in both cardiac and renal injury. This metabolic flux shifts are also termed energy metabolism remodeling (Razeghi et al., 2002) (Huang et al., 2020a). Growing evidences, including ours, have suggested damaged mitochondria as a vital contributor to energy metabolism remodeling in CRS (Huang et al., 2020a) (Mapuskar et al., 2019; Tirichen et al., 2021; Shi et al., 2022). Mitophagy is an essential machinery for the removal of damaged mitochondria, maintaining mitochondrial quality control and cellular homeostasis (Pickles et al., 2018). Mitophagy was activated by TAT-Beclin 1 to alleviate HFD-induced mitochondrial dysfunction, myocardial lipid accumulation, and diastolic dysfunction (Tong et al., 2019). Damaged mitochondria initiate mitophagy to eliminate impaired mitochondria and ROS, maintaining energy metabolism (Pickles et al., 2018). Further studies have shown that impairment of mitophagy induces mitochondrial dysfunction and lipid accumulation, thereby exacerbating diabetic cardiomyopathy (Shao et al., 2020). In addition, high fat diet (HFD) could suppress mitophagy activity and cause damaged mitochondria to accumulate in the heart, while acetyl coenzyme A carboxylase 2 (ACC2) knockout-mediated increased FAO prevents HFD-induced cardiomyopathy by the maintenance of mitochondria function *via* enhancing Parkin-mediated mitophagy (Jones and Sies, 2015). Therefore, modulating mitophagy may alleviate CRS4 *via* restoring energy metabolism modeling.

### 4.8 Mitophagy in cardiorenal syndrome type 4

Studies have shown that CKD is an independent prognostic risk factor for CVD, and renal insufficiency significantly increases mortality in patients with HF (Inaguma et al., 2017). The main pathological changes of kidney in CKD include glomerulosclerosis, tubular atrophy, interstitial fibrosis and inflammatory cell infiltration (Webster et al., 2017a; Wang X. H. et al., 2022), while a growing body of evidence observes cardiac interstitial fibrosis, cardiomyocyte hypertrophy, and mitochondrial swelling and damage in the heart (Bigelman et al., 2018). Of note, mitochondrial injury has been identified as the common pathological change in both kidney and heart in the state of CKD (Shi et al., 2022). Recent studies have reported that modulating mitophagy may reverse the above pathological changes in the injured kidney and heart through eliminating damaged mitochondria and maintaining mitochondrial homeostasis (Quiles and Gustafsson, 2022), hinting that mitophagy might link these pathological changes between the heart and kidney.

Further, we speculate that the underlying mechanisms are as follows. First, damaged kidney releases pro-inflammatory factors or uremic toxins into the circulation (Brennan et al., 2021; Ravid et al., 2021), such as indoxyl sulfate (IS), high phosphate (HP), and p-Cresyl Sulfate (PCS), which can directly damage the mitochondria of the heart and regulated mitophagy. Second, kidney-derived soluble biomolecules or proteins, such as C-X3-C motif chemokine ligand 1 (CX3CL1), apolipoprotein A1, albumin, the tumor necrosis factor superfamily member 14 (TNFSF14), circulate to cardiomyocytes, binding to the receptors or adapters on the surface of cardiomyocytes, thus affecting the mitophagy of cardiomyocytes (Zhao et al., 2019; Zheng C. et al., 2022). Mitochondria may sense these various stimuli in the extracellular environment and respond to heart (kidney)-derived biomolecules *via* the activation or inhibition of mitophagy. Finally, impaired mitophagy leads to insufficient ATP synthesis, which further exacerbates oxidative stress, inflammation and apoptosis, ultimately forming a vicious cycle and leading to pathological damage to the heart and kidney. Targeting mitophagy might break the crosstalk and improve the pathophysiological changes of CRS. These results suggest the vicious circle between kidney and heart may be mediated by impaired mitophagy.

In view of the complex process and mechanism of mitophagy, targeting mitophagy is currently controversial in different disease models (Li et al., 2021). Some studies have reported that enhancing mitophagy plays a protective role in atherosclerosis, as well as HF (Abudureyimu et al., 2020; Poznyak et al., 2021; Duan et al., 2022). On the contrary, other studies suggest that promoting mitophagy can accelerate AS progression and HF (Li et al., 2018; Zhang et al., 2020). Simultaneously, mitophagy might play distinct roles in the progression of different disease stages. For instance, mitophagy improves cardiac function and exerts a protective effect on HF. However, as the disease progresses, cardiac remodeling decompensates, and excessive mitophagy leads to unnecessary protein degradation, thereby accelerating the development of HF (Kostin et al., 2003). Hence, timely and appropriate regulation of mitophagy to maintain mitochondrial homeostasis of cardiomyocytes can serve as potential therapeutic approaches against CKD, which still requires more basic experiments and clinical studies to verify.

Moreover, in the pathological process of CRS4, the specific mechanism leading to the activation of mitophagy remains unclear. And the functional role of mitophagy in different cell type of kidney and heart remains elusive. For example, promoting mitophagy in VSMCs accelerates atherosclerosis progression (Kostin et al., 2003; He et al., 2019), whereas in endothelial cells and macrophages, promoting mitophagy exerts an atheroprotective role (Li P. et al., 2020; Choi et al., 2021; Jin Y. et al., 2022). Besides, although enhanced mitophagy in rat ventricular myoblast cells (H9c2) has been demonstrated to provoke HF (Huo et al., 2021), it has also been reported that the promotion of mitophagy in murine cardiac myocyte cell line (HL-1) prevents the development of HF. These controversial results further confirm that the regulation of mitophagy levels in distinct cell types may determine the progress and fate of disease. Comprehensive understanding of the explicit function and regulatory mechanisms of mitophagy in different cell types in CRS4 will be beneficial for developing new therapeutic strategies for CRS4. The mechanistic studies and clinical trials are urgently required to gain a deeper understanding of the efficacy and safety of these potential treatments.

## 5 Mitophagy-targeted therapy

In view of the pivotal role of mitophagy in CRS4, pharmacological modulation of mitophagy may serve as a promising strategy for the prevention and treatment. Pharmacological enhancement of mitophagy is beneficial in cardiac and renal disease models, including renal fibrosis, DKD and HF (Ma et al., 2021) (Liu B. et al., 2022) (Han et al., 2021) (An et al., 2021). Therefore, the potential therapy aimed at modulating mitophagy may be a promising treatment strategy against CRS4 (Table 1).

**TABLE 1:** Potential treatments targeting mitophagy in CRS4.

Potential treatments	Key findings	References
Clinical drugs	ARB: Alleviate left ventricular hypertrophy	Zhang et al. (2014)
	Statins: Inhibit cardiomyocyte apoptosis and improve CKD by reducing inflammation and oxidative stress	Hsieh et al. (2019)
	Metformin: Relieve myocardial injury in Adriamycin-induced heart injury and protect human renal epithelial cells from high glucose-induced apoptosis	Zhao and Sun, (2020)
		Arinno et al. (2021)
	BMSCs: Inhibit apoptosis and pyroptosis to ameliorate SI-AKI.	Guo et al. (2021)
Stem cells	TUDCA: Improve the functional recovery, including kidney recovery, limb salvage, blood perfusion ratio, and vessel formation	Yoon et al. (2019)
	MSCs: Exhibit lower levels of urinary albumin-to-creatinine ratio, less mesangial expansion, higher number of podocytes	Savio-Silva et al. (2021)
	Ameliorate mitochondrial dysfunction, apoptosis in endothelial cells in diabetic rats	Zhu et al. (2018)
	UMI-77: Ameliorate renal fibrosis in UUO mice	Jin et al. (2022a)
Small molecule agents	MitoTEMPO: Ameliorate renal function and podocytes injury in CKD rats	Liu et al. (2022a)
	AKG: Inhibit pressure overload-induced myocardial hypertrophy and fibrosis and improved cardiac systolic dysfunction	An et al. (2021)
	JQ1: Improve mitochondrial function, and repair the cardiac structure and function of the diabetic heart	Mu et al. (2020)

### 5.1 Clinical drugs

#### 5.1.1 ACEIs

A meta-analysis reported that monotherapy or the combination of an angiotensin-converting-enzyme inhibitor (ACEI) and an angiotensin II receptor blocker (ARB) was one of the most effective strategies against end-stage renal disease (ESRD) (Palmer et al., 2015). It has been reported that the ARB drug, valsartan, relieved left ventricular hypertrophy, improved myocardial autophagy and mitophagy, and increased mitochondrial biosynthesis to relieve unilateral renovascular hypertension in pigs (Zhang et al., 2014). ACEIs drugs are also strictly limited for clinical use in patients with renal insufficiency, anuric renal failure and CKD patients with serum creatinine levels greater than 225 μmol per liter (Ferrario et al., 2022).

#### 5.1.2 Statins

The existence of a causative role of lipids in the pathogenesis of proteinuria and kidney disease has been demonstrated by both clinical and experimental studies (Vaziri, 2016) (Herman-Edelstein et al., 2014) (Hao and Breyer, 2007). Statins were demonstrated to be effective for cardiovascular protection in predialysis CKD patients (Sharp Collaborative Group, 2010) (Cannon et al., 2015) (Wahl et al., 2016). As reported, statins, such as simvastatin, promoted mitophagy against HF *via* increasing PINK1 and Parkin protein expression *in vivo* (Hsieh et al., 2019). Further, the SHARP trial (Sharp Collaborative Group, 2010) demonstrated that lowering LDL-cholesterol level using simvastatin plus ezetimibe reduced the incidence of major atherosclerotic events in patients with CKD. These results were reinforced by outcomes of the subsequent IMPROVE-IT study (Cannon et al., 2015). Emerging evidence suggests that pitavastatin triggers mitophagy though the calcium-dependent CAMK1-PINK1 pathway to promote endothelial progenitor cell (EPC) proliferation and retard the progression of atherosclerosis, hinting that statins may hold the potential therapeutic effect on the treatment of CRS4 (Yang et al., 2022).

#### 5.1.3 Metformin

As reported, metformin, a classic anti-diabetic drug, restored Parkin-mediated mitophagy by activating protein phosphatase 2A (PP2A) to inhibit nuclear factor kappa B (NF-κB), thereby protecting human renal epithelial cells from high glucose-induced apoptosis (Zhao and Sun, 2020). Notably, another study found metformin significantly relieved myocardial injury in Adriamycin-induced cardiac injury by reducing the excessive mitophagy, oxidative stress and inflammation (Arinno et al., 2021). These studies indicate that metformin exerts different effects on mitophagy in different cell models, which may be due to the bidirectional regulation of mitophagy by metformin, but the ultimate effect is to protect cells against damage (Arinno et al., 2021). Metformin is recommended as a first-line treatment for type 2 diabetes because of its safety, low cost, and potential cardiovascular benefits (Tanner et al., 2019). However, the use of metformin was restricted in CKD patients due to concerns about drug accumulation and metformin-related lactic acidosis. Although there is currently insufficient evidence to support the safety of metformin in patients with eGFR values below 30 ml/min/1.73 m^2^, metformin is generally recommended to be discontinued, when renal function falls below this level.

#### 5.1.4 Stem cells

Mesenchymal stem cells (MSCs) are one of the most promising cell sources for curing kidney diseases due to their self-renewal and multi-directional differentiation potential in regenerative medicine research (Zhou and Glowacki, 2017). Previous studies have reported that MSCs transplantation showed therapeutic effects in heart-kidney injury models (Yuan et al., 2021) (Ishiuchi et al., 2020) (Jin et al., 2019) (Tseng et al., 2021) (Liao et al., 2019) (Cai et al., 2016). MSCs could migrate to the damaged kidney tissues of rats, and then upregulate SIRT1-Parkin axis to promote mitophagy in renal tubular epithelial cells, thereafter alleviating sepsis-induced AKI (Guo et al., 2021). In mice with CKD-associated hindlimb ischemia, tauroursodeoxycholic acid (TUDCA) protected mitochondrial membrane potential and restored mitochondrial dysfunction in CKD-HMSCs by activating mitophagy. Recently, it has been reported that uremic toxin P-Cresol (PC) can impair the function of MSCs, while pioglitazone can up-regulate the expression of PINK1 to activate mitophagy and protect MSCs from PC-induced mitochondrial dysfunction (Lee et al., 2018). In CVDs, MSCs have been reported to activate mitophagy and significantly alleviate mitochondrial dysfunction as well as apoptosis though inhibiting the expressions of Parkin, PINK1 and TFAM in human umbilical vein endothelial cells (HUVEC) exposed to high glucose (Savio-Silva et al., 2021) (Zhu et al., 2018). These results collectively suggest that stem cells can be used as a potential therapy strategy for CRS, which needs further experimental investigations and clinical trials. Although stem cells have a good therapeutic prospect in the treatment of CRS, due to the complex source of stem cells, the preparation process and quality control of stem cells cannot be controlled, which affects the safety and effectiveness of stem cell therapy (Sivanathan and Coates, 2020). Moreover, stem cells may have potential carcinogenicity, which greatly limits their clinical applications (Hickson et al., 2016). Emerging evidence confirms that MSC-exosomes can be used as natural carriers for targeted drug delivery (Cao et al., 2022). Thus, therapeutic agents can be efficiently incorporated into exosomes and then delivered to diseased tissues. In addition, MSC exosomes contain biologically active substances, such as proteins, messenger RNAs and microRNAs (Suzuki et al., 2016; Yea et al., 2021). Therefore, MSC-exosomes have emerged as a promising cell-free therapy for CVD.

#### 5.1.5 Small molecule agents

Small molecule agents mainly refer to some chemical synthetic compounds with molecular weight less than 1000. The structure of small molecule agents has good spatial dispersion, and its chemical properties determine its performances and pharmacokinetic properties. These characteristics make small molecule agents show great advantages in CRS and other complications.

As reported, UMI-77 enhanced mitophagy, reversed mitochondrial damage, reduced ROS production, and inhibited renal fibrosis in UUO mice (Jin L. et al., 2022). In addition, mito-TEMPO, a mitochondrial targeting antioxidant, activated PINK1-Parkin pathway to induce mitophagy *in vivo* and *in vitro*, which significantly ameliorated the podocyte injury in a rat model of CKD (Liu B. et al., 2022). Another small molecule metabolite of tricarboxylic acid (TCA) cycle, α-ketoglutarate (AKG), promoted mitophagy to eliminate damaged mitochondria, mitigated myocardial hypertrophy and fibrosis caused by pressure overload, and improved cardiac systolic dysfunction during cardiac injury (An et al., 2021). It has also been reported that the up-regulation of BRD4 in the heart of diabetic mice inhibited PINK1-Parkin-mediated mitophagy, while the selective bromine domain inhibitor JQ1 inhibited the expression of BRD4, activated PINK1-Parkin-mediated mitophagy, and subsequently reduced the accumulation of damaged mitochondria to alleviate cardiac structural and functional damage (Mu et al., 2020). These results suggest that small molecule agents are promising and effective strategies for CRS. Small molecule agents also have limitations in their application, including poor bioavailability, lack of tissue targeting and safety evaluation (Uddin et al., 2021). Therefore, a comprehensive evaluation of safety for clinical applications is necessary. Secondly, advanced drug delivery systems such as nanoparticle-mediated drug delivery and molecular structure optimization to increase bioavailability and tissue targeting are also required. We believe that small-molecule agents remain a promising therapeutic agent by optimizing the structure and improving the above shortcomings.

## 6 Conclusion and prospect

Mitophagy plays a vital role in mitochondrial homeostasis in multiple diseases, particularly CKD, CVD, and CRS4. In this review, we discussed the complex interaction between the heart and kidney as well as the function of mitophagy during this process. In addition, general clinical drugs, stem cells and small molecule agents are considered as feasible therapeutic strategies targeting mitophagy to retard the progression of CRS4. Therefore, delicate modulation of mitophagy may serve as a novel therapeutic strategy against CRS4. Further, it cannot be ignored that excessive mitophagy may also be harmful and ultimately result in cell death, even if the loss of mitophagy is deleterious to mitochondrial homeostasis. In developing drugs and small-molecule agents for mitophagy, how to modulate the targeting of mitophagy is still an urgent problem to be solved. The development of nanomaterials targeting kidney and heart may be a promising direction for the treatment of CRS4. Simultaneously, the efficacy, specificity, and sensitivity of natural inducers of mitophagy have not been demonstrated in human CRS, and therefore future clinical trials are urgently required. Despite these limitations, the current experimental and clinical findings collectively indicate that targeting mitophagy might be a promising therapeutic approach for those patients with CRS4.
